# Evaluation of the *in vitro* activity of synthetic derivatives of N-cyclohexyl-3-(3-methylphenyl)-1,2,4-oxadiazole-5-amine on the strain y of *Trypanosoma cruzi* and an *in vivo* toxicity study

**DOI:** 10.1590/0074-02760250289

**Published:** 2026-07-20

**Authors:** Yasmim Mendes Rocha, Lyanna Rodrigues Ribeiro, Gabriel Acácio de Moura, Marlos de Medeiros Chaves, João Pedro Viana Rodrigues, Emanuel Paula Magalhães, Sara Ingrid Caetano Gomes Barbosa, Lucas Soares Frota, Selene Maia de Morais, Wildson Max Barbosa da Silva, Valentina Nascimento e Melo de Oliveira, Ronaldo Nascimento de Oliveira, Alice Maria Costa Martins, Roberto Nicolete

**Affiliations:** 1 Universidade Federal do Ceará, Programa de Pós-Graduação em Ciências Farmacêuticas, Fortaleza, CE, Brasil; 2 Fundação Oswaldo Cruz-Fiocruz, Eusébio, CE, Brasil; 3 Universidade Estadual do Ceará, Programa de Pós-Graduação em Biotecnologia, Laboratório de Química de Produtos Naturais, Fortaleza, CE, Brasil; 4 Universidade Estadual do Vale do Acaraú, Sobral, CE, Brasil; 5 Universidade Federal Rural de Pernambuco, Departamento de Química, Recife, PE, Brasil; 6 Instituto Federal de Educação, Ciência e Tecnologia, Ipojuca, PE, Brasil

**Keywords:** Trypanosoma cruzi, Chagas disease, oxadiazoles, antiproliferative effect

## Abstract

**BACKGROUND:**

Chagas disease (CD) is caused by *Trypanosoma cruzi*. Treatment is based on benznidazole (Bz), although it has significant limitations, such as low efficacy in the chronic phase. Therefore, the search for new therapies with greater selectivity and antiparasitic activity is necessary. In this context, 1,2,4-oxadiazole stands out for its biological properties, including antiparasitic activities.

**OBJECTIVE:**

To evaluate the *in vitro* activity of N-cyclohexyl 3-(3-methylphenyl)-1,2,4-oxadiazol-5-amine derivatives against the Y strain of *T. cruzi* and an *in vivo* toxicity study.

**METHODS:**

Cytotoxicity was evaluated in LLC-MK2 cells by the MTT assay, while the antiparasitic effect on the three *T. cruzi* life forms was determined by counting. Flow cytometry analyses were then conducted to investigate possible death pathway mechanisms and antioxidant and antiacetylcholinesterase activities. Scanning electron microscopy (SEM) was also performed to observe morphological changes caused by the compounds. Finally, *in vivo* acute toxicity tests were performed on ZebraFish embryos.

**RESULTS:**

The results presented show distinct cellular toxicity profiles in LLC-MK2 cells, in addition to demonstrating antiparasitic activity at different concentrations. In amastigotes, cytotoxic effects were stimulated. The molecules also induced an increase in reactive oxygen species and membrane damage, in addition to loss of integrity and morphological changes. The antioxidant activity revealed a high capacity for scavenging free radicals, suggesting an alteration of the redox balance of the parasite, in addition to showing inhibition of acetylcholinesterase, an important enzyme present in the formation of parasites, which choline is a constituent. In the ZebraFish model, molecule 2a showed dose-dependent embryonic toxicity, with an LC50 of 14-15 µM.

**MAIN CONCLUSIONS:**

The calculated conclusions appear to indicate an antiparasitic effect associated with cell death mechanisms. However, further studies are needed to reduce toxicity in the animal model and increase delivery to the site of action.

Chagas disease (CD) is an infection caused by *Trypanosoma cruzi*, affecting approximately 6 million people in the Americas, including Bolivia, Argentina, Brazil, and Colombia, as well as the United States and Europe.^1^ ^,2^ In most cases, the disease is asymptomatic or presents with mild, nonspecific symptoms.

The acute phase ends when the parasitaemia disappears and the remaining parasites reside, in most cases, in the deep tissues.^3^ Finally, the chronic phase manifests as a multisystemic disease that primarily affects the cardiovascular and digestive systems.^4^ There are only two medications that demonstrate proven efficacy against *T. cruzi* infection, which include benznidazole (Bz) and Nifurtimox (Nfx), both used since 1970 and administered as monotherapy for 60 days.

Nifurtimox treatment has frequent adverse effects, including anorexia, nausea, vomiting, and polyneuropathy, and is therefore discontinued in up to 75% of cases.^5^ While Bz remains the first-choice medication option, it may present adverse effects such as contact dermatitis, skin rash or photosensitisation.

Although Bz has guaranteed efficacy in the acute phase of the disease, it has a poor long-term tolerability profile, in addition to ineffectiveness in the chronic phase and lack of cure in chronically infected adults.^6^ This highlights a clear and substantial priority for new, better, and safer antiparasitic drugs for CD that demonstrate trypanocidal action and greater selectivity against parasites.

The compound oxadiazole is a five-membered heterocyclic compound containing two nitrogen atoms and one oxygen atom. It is part of an aromatic linking group capable of connecting to a variety of substituents and exhibits the same biological activity as esters, amides and carbamates, and therefore, it behaves as a bioisostere, with better hydrolytic and metabolic properties.^7^

1,2,4-Oxadiazole has been frequently studied due to its important biological activities, such as antibacterial, anti-inflammatory, antituberculous, antifungal, antioxidant and antiparasitic.^8^

The aim of this study was to evaluate the effects of three synthetic 1,2,4-oxadiazole derivatives on the life cycle forms of the parasite, as well as its cytotoxicity, cell killing activities, morphological alterations, and antioxidant activities.

Therefore, the target product must be at least as effective and better than the current treatment in terms of safety and clinical outcomes.

## MATERIALS AND METHODS

**Fig. 1:**
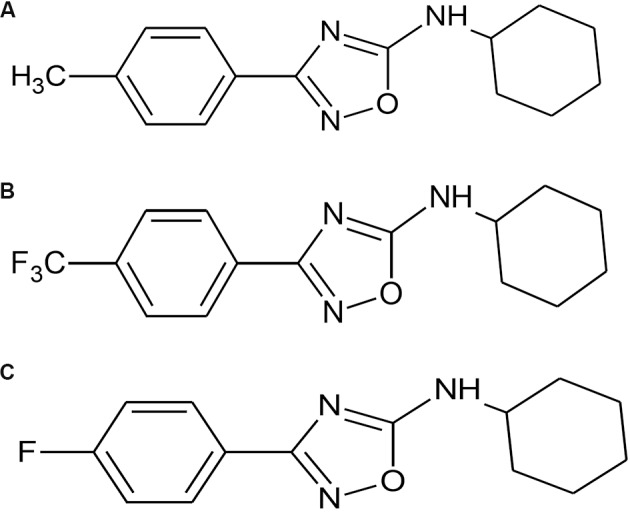
chemical structures of molecules 2a, 2f and 2i. Molecules derived from oxadiazole. In (A) molecule 2a, in (B) molecule 2f, and in (C) molecule 2i.

*Chemicals* - The N-cyclohexyl-3-(3-methylphenyl)-1,2,4-oxadiazol-5-amine derivative was synthesised from a solution of arylamidoximes and dicyclohexylcarbodiimide (DCC 1) in DMF for 10 min under microwave irradiation by the Laboratório de Síntese de Compostos Bioativos of the Universidade Federal de Pernambuco.^8^ It was screened the 1,2,4-oxadiazole derivatives, which were designated as molecules 2a, 2f, and 2i (Fig. 1). Furthermore, these molecules were diluted in sterile dimethyl sulfoxide (DMSO) to obtain stock solutions at 0.2 M concentrations.

Benznidazole, used as a reference drug and positive control in our assays, was donated by the Laboratório Farmacêutico de Pernambuco (LAFEPE). For the assays, serial dilutions were performed using a sterile phosphate buffer solution (PBS). All substances were diluted in sterile DMSO to provide stock solutions at a concentration of 0.2 M, in order to obtain working solutions, such that the DMSO concentration did not exceed 0.5%.

*Cytotoxicity assay* - The cytotoxicity of the oxadiazole derivatives (2a, 2f, and 2i) was evaluated to assess their selectivity for *T. cruzi* using the 3-(4,5-Dimethyl-2-thiazolyl)-2,5-diphenyl-2H-tetrazolium bromide (MTT) in LLC-MK2 cell line (monkey renal epithelial cells), obtained from the Rio de Janeiro Cell Bank, as previously described.^9^ Briefly, the cells were cultured in Dulbecco's Modified Eagle Medium (DMEM).

After this period, the experimental groups were incubated with the MTT solution (2.5 mg/mL), and after 3 h, the formazan salt was solubilised by the addition of DMSO.

The plates were incubated for 17 h, and their absorbance was read at 570 nm to obtain the percentage of viable cells compared to the control group. Cell viability percentages were estimated using the concentration required to reduce 50% of cell viability (CC50 µM).

*Antiparasitic effect* - The epimastigote forms (Y strain) of *T. cruzi* were provided by the Trypanosomatid Collection of the Instituto Oswaldo Cruz, Fiocruz, Rio de Janeiro, Brazil. The parasites were cultured in liver infusion tryptose (LIT) medium, with the addition of antibiotics and supplemented with 10% foetal bovine serum (FBS).

At a concentration of 1 x 10^6^ cells/mL, the cultures were seeded in 96-well plates and treated with the derivatives (2a, 2f, and 2i) and Bz at different concentrations (100-1.5 µM).

Untreated cells were used as a negative control. The antiproliferative effect was quantified by counting parasites using the Neubauer chamber and the percentage of viable cells when compared with untreated cells was used to estimate the concentration capable of inhibiting the proliferation of epimastigote forms by 50% (IC50).

*Evaluation of the effect on trypomastigote forms* - After host cell infection, trypomastigote forms were obtained at a concentration of 1 x 10^6^ trypomastigotes/mL and maintained in DMEM medium with 10% foetal calf serum (FCS).

At this concentration, the parasites were incubated for 24 h with the study molecules and Bz in 96-well plates. Untreated parasites were used as a negative control and considered 100% viable, and 0.5% DMSO was used as a vehicle.

The percentage of cell viability was calculated to estimate the lethal concentration for 50% of the trypomastigotes (LC50). Furthermore, the selectivity index (SI) of the trypomastigote forms relative to the host cells (CC50/LC50) was calculated.

The counting of trypomastigote forms in the Neubauer chamber was performed after 24 h, considering that these forms do not multiply *in vitro* without host cells and, therefore, begin to die naturally after this period.^10^

*Evaluation of the effect on amastigote forms* - To evaluate the effect of the derived molecules on intracellular amastigote forms, 1 x 10^5^ LLC-MK2/mL were cultured in 24-well plates and incubated for 24 h at 37ºC.

Soon after, the trypomastigote forms (1 x 10^6^) were added to LLC-MK2 cells in DMEM medium with 2% FBS and incubated again for 48 h. After this period, the medium was changed and the wells were treated at concentrations of 30 and 60 µM with the study molecules (2a, 2f, and 2i) and Bz for 24 h.

Subsequently, the slides were fixed with Bouin's solution and stained with Giemsa and after 24 h, the amastigote forms internalised in each infected and uninfected cell were counted, as well as the number of amastigotes/100 infected cells and the survival index, which evaluates the capacity of a molecule to eliminate parasites from a host cell.^11^

*Flow cytometry assays* - Flow cytometry was used to evaluate the profile and mechanisms of cell death caused by the study molecules. Epimastigotes were used at a concentration of 1 x 10^6^ and treated (100 and 50 µM) for 24 h.^12^

*Evaluation of cell death mechanisms* - The mechanisms of cell death using the fluorescence markers Annexin V-phycoerythrin (V-PE) and 7-ActinomycinD (7-AAD) were evaluated with characteristics indicative of cell death by apoptosis and necrosis, respectively, using epimastigote forms (1 x 10^6^) induced by the study molecules (100 and 50 µM) for 24 h.

After this period, the cultures were washed with PBS (1x) and with binding buffer (2x), containing 10 mM Hepes, 140 mM NaCl, 2.5 mM CaCl_2_ and pH 7.4.

Then, 100 µL of the binding buffer and 10 µL of the markers were added, according to the manufacturer's instructions (Annexin V/PE Apoptosis Detection Kit I, BD Bioscienses). The experimental groups were analysed using FL2 and FL3 detectors, orange and red fluorescence, for AvPE and 7-AAD, respectively.

At least 10,000 events were performed and divided into four quadrants: viable cells, with low staining for both dyes; necrotic cells, with staining for 7-AAD; apoptosis, with high staining for AvPE; and double-labelled cells. If loss of membrane integrity or externalisation of phosphatidylserine occurs, it is due to staining with 7-AAD or AvPE, respectively.^12^

*Assessment of cytoplasmic reactive oxygen species (ROS) production* - This assay is based on the investigation of cytoplasmic oxidative stress using the fluorescence marker 2'7'-dichlorofluorescein diacetate (DCFH-DA).

Gating strategy: unstained cells were used as a negative control, while cells treated with 50 µM of Lupeox were used as a positive control to induce cytoplasmic oxidative stress.

Epimastigote forms (1 x 10^6^) were plated in 24-well plates, using the study molecules (100 and 50 µM) for 24 h. Then, 10 µL of the DCFH-DA solution was added and incubated for 3 h.

The plate was kept in the dark until the end of the treatment and after 24 h of incubation, the cells were centrifuged and finally analysed by flow cytometry (10,000 events per sample) in the FACSCalibur equipment.^12^

*Assessment of mitochondrial transmembrane potential (ΔΨm) determination* - Finally, to assess changes in mitochondrial transmembrane potential induced by the study molecules, a lipophilic and cationic dye called Rhodamine 123 (Rho123), a marker that emits red fluorescence, was used.^13^

Gating strategy: unstained cells were used as a negative control, while cells treated with 50 µM of rotenone were used as a positive control to induce changes in mitochondrial transmembrane potential.

The epimastigote forms (1 x 10^6^) were incubated with the study molecules (100 and 50 µM) for 24 h. After this incubation period, 10 µL of Rho123 was added for 30 min. The samples were centrifuged, washed twice with PBS, and resuspended in PBS (500 µL/tube). Finally, they were analysed by flow cytometry.

The results were expressed according to the fluorescence intensity, using the FL2 detector.^12^

*Antioxidant and antiacetylcholinesterase activities in vitro* - Antioxidant activity was evaluated by the DPPH (2,2-diphenyl-1-picrylhydrazyl) method following the methodology described by Becker et al.^14^ with modifications, and by the ABTS (2,2'-azino-bis(3-ethylbenzothiazoline-6-sulfonic acid)) method as described by Re et al.^15^

Both tests were conducted in a 96-well flat-bottom microplate using a BioTek ELISA reader, model ELX 800.

The inhibitory activity of recombinant acetylcholinesterase (AChE) enzyme was measured in 96-well flat-bottom plates using a BioTek ELISA reader, model ELX 800, with the "Gen5 V2.04.11" software, based on the methodology described by Rhee et al.^16^ and Trevisan et al.^17^

All solutions were used as negative standards, except for the sample. All samples were analysed in triplicate.

*Scanning electron microscopy (SEM) analysis* - To analyse the morphological changes induced by the study molecules, the epimastigote forms were incubated (1 x 10^6^) for 24 h with the study molecules and Bz (50 and 25 µM). Glutaraldehyde (2.5%) was then added to each sample.

The fixed samples were exposed to increasing amounts of 30, 50, 70, 90, and 100% ethanol for dehydration, followed by centrifugation at 800g for 5 min. After this process, the slides were coated with gold layers (20 nm thick) using a QT150-ES-Quorum Metallizer and finally analysed using a Quanta 450 FEG-FEI SEM.^12^

*Acute toxicity analysis in ZebraFish embryos* - To explore the *in vivo* testing, acute toxicity analyses were performed on ZebraFish embryos, according to the Organisation for Economic Co-operation and Development (OECD) FET236, conducted in accordance with study plan ZS-TOX-06/25-1.

WT ZebraFish (*Danio rerio*) embryos were obtained through natural spawning of breeding stock, according to the breeding protocol for the progenitors. The animals were kept in a recirculating water system (density of 2 fish/L), with water quality maintained by mechanical, biological, and chemical filtration, and passed through a UV light sterilisation unit.

Water chemical parameters were maintained at pH ~7, temperature 27ºC, oxygen ~6 mg/L, total ammonia < 0.01 mg/L, and conductivity of 600 μS/cm. The photoperiod was set at 14 h/12 h, with the light phase beginning at 7:00 am (190 lux). Fish (juveniles and adults) were fed brine shrimp (*Artemia* sp., *Artemia salina* do RN®, Brazil) and commercial flake food (Alcon Basic®, Brazil; 60% protein and 15% fat). Food was offered three times a day.

Adult fish were placed in breeding tanks (three males: two females) containing a removable, transparent partition with holes separating males and females, but allowing chemical and visual contact between individuals.

The partition was removed in the first hour of light (07:00 h) and spawning occurred naturally. One hour after spawning, embryos were collected, checked for fertilisation (under a stereomicroscope at 40x magnification; MOTIC), and counted.

The percentage of fertilised eggs (> 70%) was verified by visual identification of the blastula stage at 3 hpf. The coagulation percentage of the negative control group (< 10%) was also verified. Each item was weighed to prepare 50 mL of the highest concentration. After preparing the stock solution, dilutions were performed to obtain the concentrations of the study molecule (2a).

Water from the recirculation system was used as a negative control. Fertilised eggs were separated, washed with system water, and transferred to 24-well polystyrene plates (1 embryo/well).

*Statistical analysis* - All statistical analyses and graphical representations were performed using R software. Data were expressed as mean ± standard error of the mean (SEM). Dose-response curves and survival analyses were generated using nonlinear regression models using the drc and ggplot2 packages. The significance level adopted was p < 0.05.

## RESULTS

*Cytotoxicity in host cells (LLC-MK2)* - The *in vitro* antiparasitic activity of the oxadiazole-derived molecules was evaluated in three parasite stages. In epimastigotes, after 24 and 48 h of treatment, the IC50 values for 2a, 2f, and 2i were 31.3 µM and 7.6 µM, 44.4 µM and 2.6 µM, 7.0 µM and 3.7 µM and for Bz (17.3 µM and 3.5 µM). In trypomastigotes, after 24 h of treatment, the LC50 values for 2a, 2f, and 2i were 14.2 µM, 2.4 µM, and 2.6 µM, and for Bz (59.1 µM), as shown in Table I.

**TABLE I:** Estimated values of CC50, IC50 and LC50 of molecules 2a, 2f and 2i and of the reference drug benznidazole (Bz)

	CC_50_ (LLC-MK2)	IC_50_ 24 h (µM)	IC_50_ 48 h (µM)	LC_50_ (µM)	SI
2a	221	31.34	7.61	14.2	15
2f	14	44.4	2.6	2.4	5.8
2i	25.7	7.0	3.7	2.6	9.8
Bz	59.1	17.3	3.5	59.1	4.7

CC50: concentration capable of causing 50% toxicity in host cells; IC50: concentration capable of inhibiting 50% of the growth of epimastigote forms; LC50: concentration capable of causing the death of 50% of trypomastigote forms; SI: selectivity index.

The SI was calculated from the CC50/LC50 (LLC-MK2 cells/trypomastigotes), and it was observed that molecule 2a appeared to be the most promising when compared to the other molecules (and even the reference drug).

**Fig. 2:**
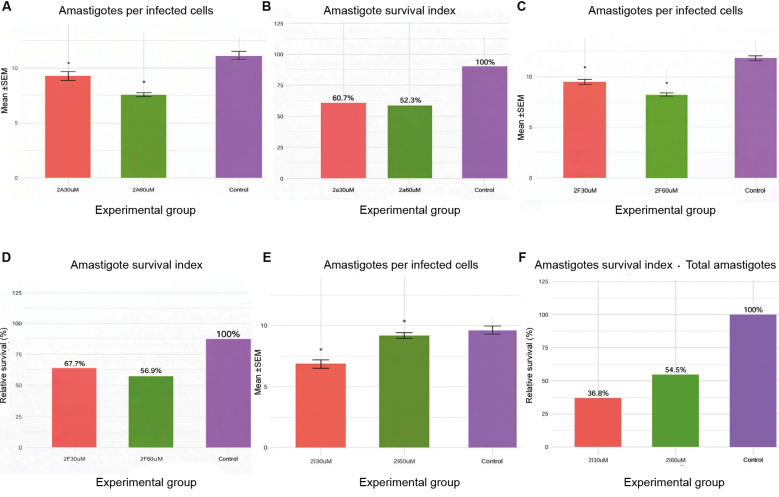
antiamastigote effect (24 h) of molecules 2a, 2f and 2i. Antiamastigote assay showing the number of amastigotes per infected cell (A, C, E) and the survival index (B, D, F) for the studied molecules 2a, 2f, and 2i, respectively. (A-B) correspond to molecule 2a, (C-D) to 2f, and (E-F) to 2i.

The antiamastigote effect is shown in Fig. 2. In items A and B, it was possible to identify a significant and expressive antiparasitic reduction in the intracellular load of amastigotes of molecule 2a compared to the control group and Bz (data not shown). The survival index was 60.7% and 52.3% for the two concentrations tested (30 and 60 μM), respectively.

In items C and D of Fig. 2, we observed that the 2f molecule showed dose-dependent activity against amastigote forms, with greater reduction of parasites at the concentration of 60 μM, suggesting a good antiparasitic effect in vitro. Furthermore, the survival index indicated moderate toxicity at 30 μM (67.7%) and increased toxicity with the dose (56.9% at 60 μM), evidencing a dose-dependent relationship for both efficacy and toxicity.

In the subsequent items (E and F), still in Fig. 2, the effect of molecule 2i is observed, which also significantly attracted its effect; however, it presented an even more important effect on the survival index results at the lowest concentration (30 μM).

*Flow cytometry assay* - The killing mechanisms identified by the flow cytometry assay were important for evaluating the killing profile of the substances involved in this study. Epimastigote forms and death markers, such as 7AAD and AxV PE, were used to evaluate the apoptotic and necrotic death pathways, respectively.

The control group (n = 10) was evaluated by quadrant analysis (UL/UR/LL/LR), with mean percentage distribution in the gated population (%) of UL 0.55 ± 0.16%, UR 3.48 ± 0.84%, LL 94.99 ± 0.90% and LR 0.98 ± 0.12% [mean ± standard deviation (SD)].

**Fig. 3:**
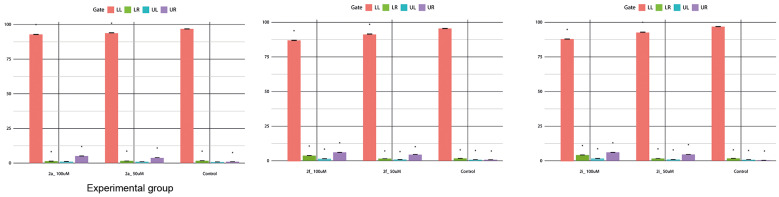
flow cytometry analysis of cell death in epimastigotes. Epimastigote forms of the *Trypanosoma cruzi* Y strain were incubated for 24 h and analysed by flow cytometry using 7-AAD and Annexin V-PE staining after treatment with molecules 2a, 2f, and 2i. Representative dot plots show the distribution of viable cells (LL), early apoptotic cells (LR), necrotic cells (UL), and late apoptotic cells (UR).

In Fig. 3, in A, molecule 2a can be seen, in the dotplot and bar quadrants, which, at the concentrations tested (50 and 100 μM), did not induce cell death by apoptosis or necrosis. The molecule's profile is virtually identical to that of the control group, with only a slight increase in late apoptosis at the higher dose.

Also in Fig. 3, in B, the graphs of the 2f molecule at a concentration of 100 μM, it is possible to see a significant increase in late apoptosis/necrosis (evidenced by the UR quadrant - purple) and a small increase in early apoptosis (LR - green), compared to the control group. However, at the lower concentration, a profile close to the control was obtained, but with a slight increase in UR.

Molecule 2i, still in Fig. 3, according to the dotplot graph in C, showed greater dispersion in the early apoptosis (LR) quadrants and more evident in late apoptosis (LR), in relation to the control.

Furthermore, the DCFH-DA marker was used to monitor the increased production of ROS in order to assess the cellular integrity profile using oxidised DCF (2'7'-dichlorofluorescein).

For ROS control with DCF, fluorescence in FL1-DCF was expressed as a geometric mean. In the controls (n = 10), the geometric mean was 11.61 ± 0.55 (min-max: 10.37-12.10), demonstrating consistency of the control.

**Fig. 4:**
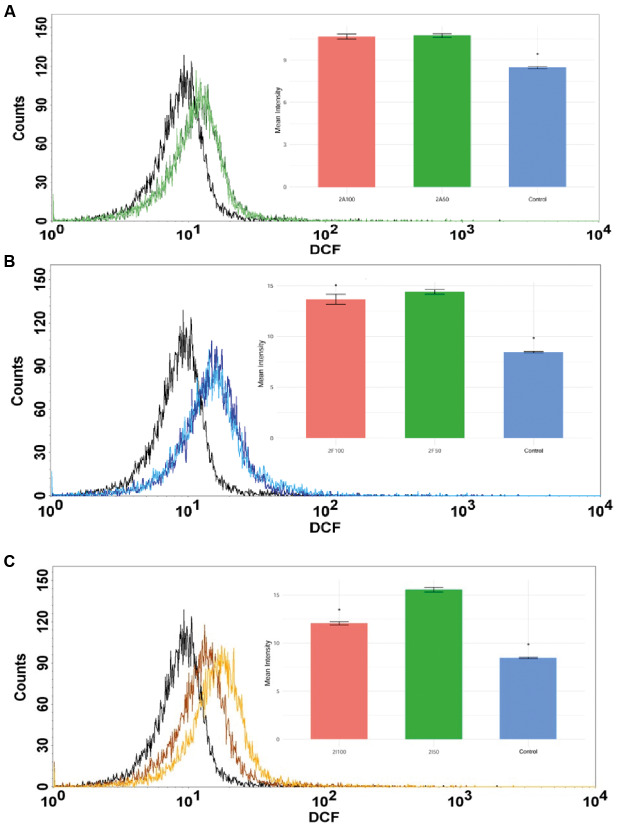
cytoplasmic reactive oxygen species (ROS) production in epimastigotes. Flow cytometry analysis of cytoplasmic ROS production in *Trypanosoma cruzi* epimastigotes treated with molecules 2a, 2f, and 2i for 24 h. Histograms show the fluorescence intensity profiles of ROS production compared to the control group (CT, black curve). For molecule 2a, dark green and light green curves represent treatments at 100 µM and 50 µM, respectively. For molecule 2f, dark blue and light blue curves represent treatments at 100 µM and 50 µM, respectively. For molecule 2i, orange and brown curves represent treatments at 100 µM and 50 µM, respectively.

According to Fig. 4, in A, flow cytometry analysis showed that treatment with molecule 2a, at concentrations of 50 and 100 μM, promoted a significant increase in the production of cytoplasmic ROS in relation to the control (p < 0.05).

According to the histogram in Fig. 4, in B, it is possible to observe a shift to the right of the fluorescence peaks; however, no significant differences are observed between the two concentrations, suggesting saturation of the pro-oxidant effect from 50 μM of molecule 2f.

The 2i molecule was also represented by flow cytometry, as shown in Fig. 4, in C, which revealed that there was a significant increase in the production of cytoplasmic ROS (in relation to the control). The largest increase was observed at 50 μM followed by 100 μM, both with a rightward shift in the DCF fluorescence histogram.

The relative reduction of ROS at 100 μM compared to 50 μM may be associated with cytotoxic effects, leading to a lower cellular capacity to generate ROS.

Furthermore, the dye Rhodamine 123 (Rho123) was also used to assess the mitochondrial transmembrane potential (ΔΨm). Mitochondrial potential monitored by Rho123 (FL1-Rho123), with fluorescence expressed as geometric mean (Geo Mean). In the controls analysed (n = 6), the average Geo Mean was 91.23 ± 0.22 (min-max: 90.90-91.48), indicating control stability.

**Fig. 5:**
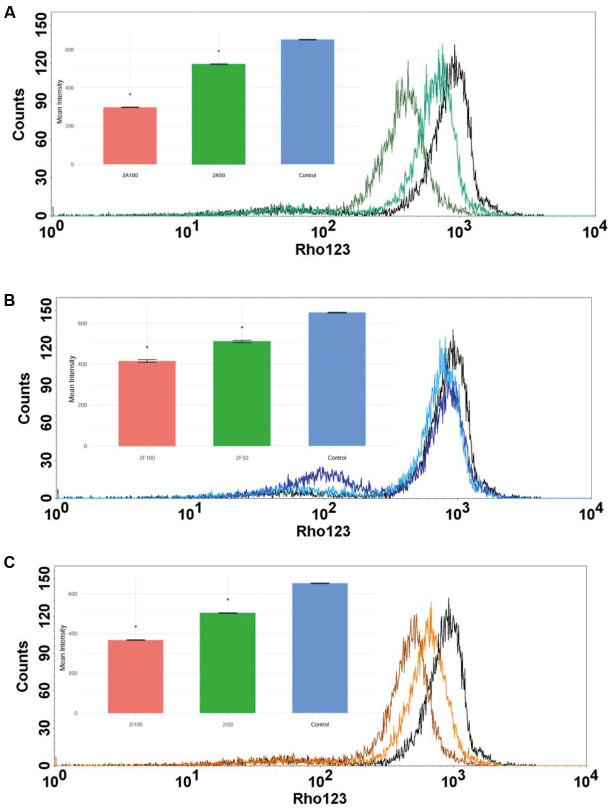
mitochondrial membrane potential (ΔΨm) evaluation in epimastigotes. Flow cytometry analysis of mitochondrial membrane potential (ΔΨm) in *Trypanosoma cruzi* epimastigotes treated with molecules 2a, 2f, and 2i for 24 h. The mitochondrial membrane potential was evaluated based on relative fluorescence intensity. In all histograms, the black curve represents the control group (CT). For molecule 2a, dark green and light green curves represent treatments at 100 µM and 50 µM, respectively; for molecule 2f, dark blue and light blue curves represent treatments at 100 µM and 50 µM, respectively; and for molecule 2i, brown and orange curves represent treatments at 100 µM and 50 µM, respectively.

Staining with the Rho123 dye showed, according to the graph above, in Fig. 5, in A, a significant and dose-dependent reduction in mitochondrial fluorescence after treatment with molecule 2a, as observed at the lowest concentration (50 μM) in relation to the highest concentration (100 μM).

The histogram shifts to the left as the dose increases, indicating depolarisation of the mitochondrial membrane potential (relative to the control group).

Meanwhile, the 2f molecule promoted a slight depolarisation of its mitochondrial transmembrane potential using Rho123, at both concentrations in a dose-dependent manner (p < 0.05 vs control). The histograms indicate a slight shift to the left with increasing concentration, shown in Fig. 5, in B.

The Rho123 assay demonstrated that the 2i molecule promoted a significant dose-dependent depolarisation of the membrane potential in cells treated with 50 μM and 100 μM, compared to the control group. Furthermore, the histogram shows a progressive shift to the left with increasing dose, reflecting lower fluorescence and loss of ΔΨm (Fig. 5, in C).

In antioxidant assays, all samples demonstrated the ability to inhibit free radicals. This potential for inhibiting oxidising radicals was evaluated by measuring the inhibition of DPPH and ABTS radicals, which are widely recognised models for assessing a substance's ability to neutralise free radicals (Table II).

**TABLE II:** Antioxidant and antiacetylcholinesterase activities of the compounds

Samples	CI_50_ DPPH• (μg.mL^-1^)	CI_50_ ABTS^+•^ (μg.mL^-1^)	CI_50_ AChE (μg.mL^-1^)
BHT (Standard)	10.25 ± 0.02	10.67 ± 0.08	-
Trolox (Standard)	12.35 ± 0.05	12.13 ± 0.07	-
Galantamine (Standard)	-	-	5.82 ± 0.02
Physostigmine (Standard)	-	-	6.68 ± 0.08
Bz (Control)	11.19 ± 0.24	16.46 ± 0.72	58.94 ± 6.48
2f	10.49 ± 0.37	12.10 ± 0.43	58.19 ± 22.32
2i	10.05 ± 0.72	13.97 ± 0.47	58.58 ± 49.13
2a	8.56 ± 0.99	17.98 ± 0.75	18.87 ± 0.30

IC50: mean inhibitory concentration; BHT: butylated hydroxytoluene; Trolox: 6-hydroxy-2,5,7,8-tetramethylchroman-2-carboxylic acid; Bz: benznidazole.

The results obtained indicate that the samples exhibited a high capacity for radical inhibition, both for the ABTS radical and for the DPPH radical, with the latter being more prominent in sample 2a, with average inhibitory concentration (IC50) values of 8.56 ± 0.99 μg.mL^-1^, being more active than commercial antioxidants BHT and Trolox.

For acetylcholinesterase inhibition, only sample 2a showed enzyme inhibition activity, being classified as having high inhibition, with an IC50 of 18.87 ± 0.30 μg.mL^-1^, showing it to be a promising molecule in the studies of inhibition of this enzyme (Table II).

*Evaluation of structural changes in the parasite* - In order to analyse epimastigote forms microscopically, based on the observation and analysis of the microstructural characteristics of materials, SEM tests were performed at two different concentrations (25 µM and 50 µM) for 24 h, along with the study molecules and the reference drug (Bz: 25 µM and 50 µM).

SEM microscopy revealed different morphologies depending on the concentration and molecule used.

**Fig. 6:**
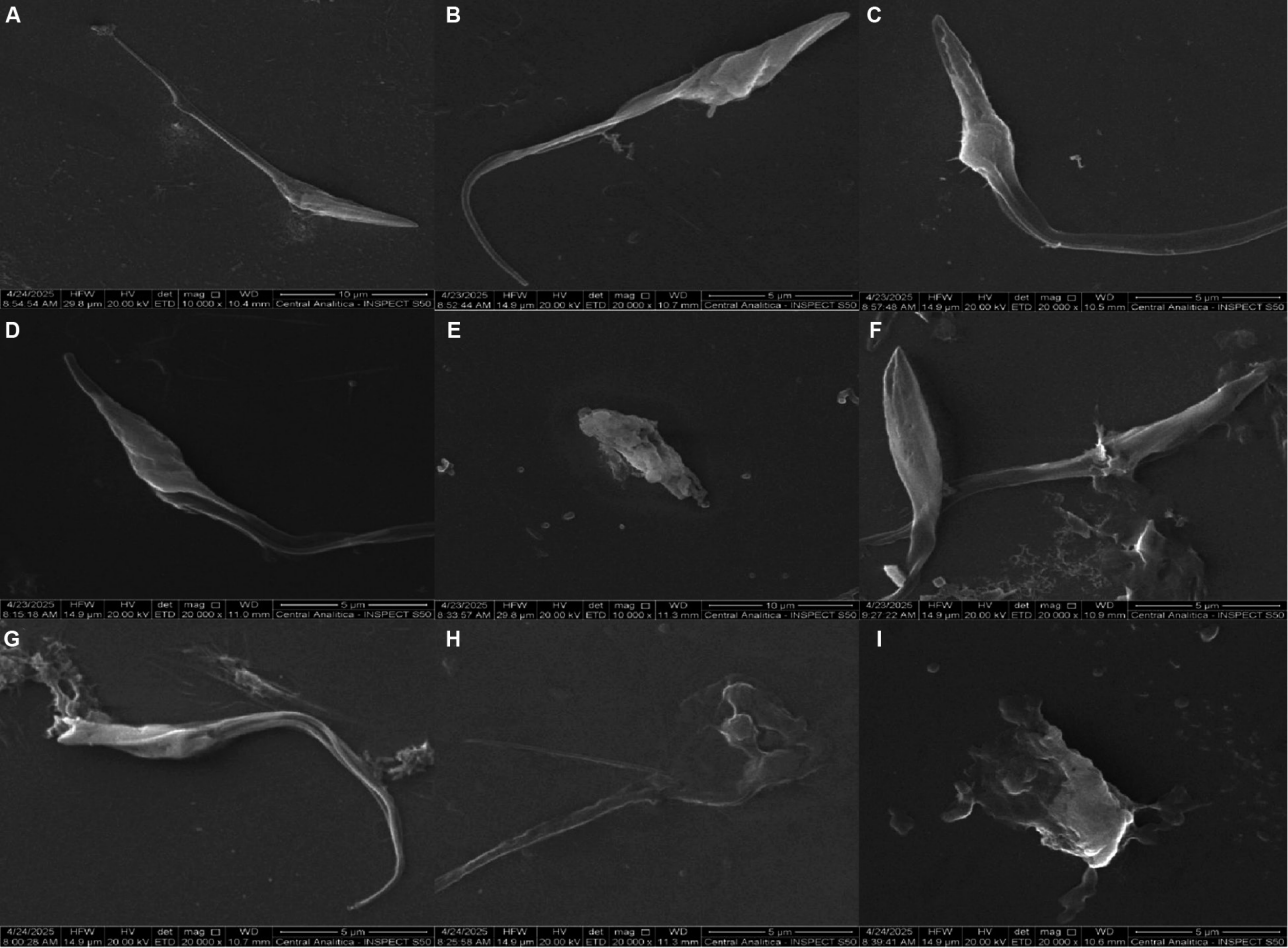
scanning electron microscopy (SEM) of epimastigotes. Representative images showing morphological alterations in *Trypanosoma cruzi* Y strain epimastigotes. In (A), the control group displays normal morphology and typical elongated shape. In (B) and (C), cells treated with benznidazole (Bz) exhibit body and tail irregularities. In (D) and (E), corresponding to treatments with molecule 2a, cells show collapsed shape and loss of typical morphology. In (F) and (G) show cells treated with molecule 2f, characterised by the presence of membrane pores. Finally, in (H) and (I), corresponding to treatments with molecule 2i, exhibit severe membrane rupture with extravasated cellular content and total loss of morphology.

In Fig. 6, in A, the control group can be seen, presenting normal morphology and typical shape, with no apparent morphological alteration. In B and C, the cells treated with Bz are observed, showing irregularity in the body and tail and membrane roughness. In D and E (molecule 2a), changes in thickened, irregular body and membrane swelling, in addition to collapsed shape, loss of shape and absence of flagellum, at the two reported concentrations, respectively.

Images F and G of Fig. 6 show cells treated with molecule 2f, in which the epimastigote forms present membrane pores, a deformed central region, and an expanded and collapsed shape with loss of morphology. Molecule 2i (items H and I) presented membrane rupture with extravasated content and total loss of morphology with the presence of vesicles/fragmented structures around it.

*Evaluation of the in vivo toxicity* - After *in vitro* assays using molecule screening (2a, 2f, and 2i), it was observed that the killing profile and SI were higher for molecule 2a. Therefore, new assays were performed with wild-type (WT) ZebraFish embryos/larvae (code: ZS-TOX-06/25-1) at concentrations of 100, 50, 25, 12.5, and 6.25 µM. The mortality rate was measured at 24, 48, 72, and 96 h.

**Fig. 7:**
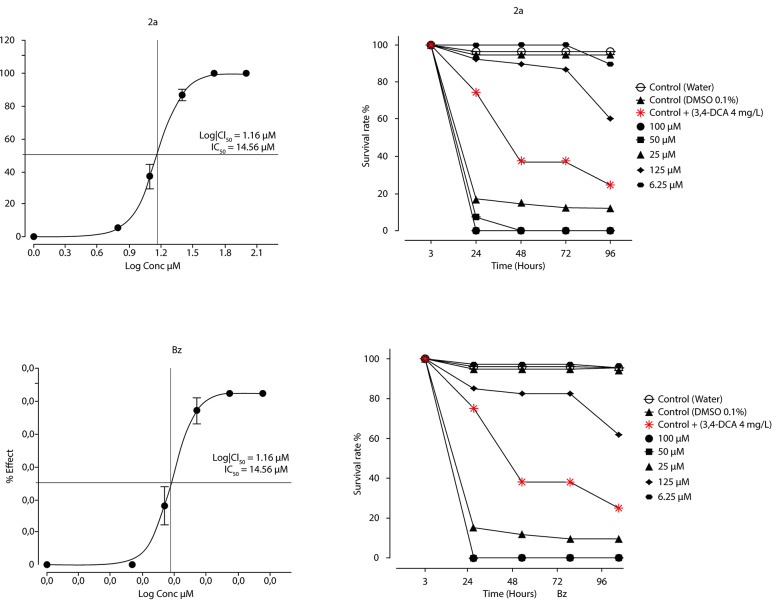
concentration effect and mortality/survival rate analysis. Analysis of the concentration-dependent effects of molecule 2a and benznidazole (Bz) on embryonic mortality and survival. Graphs (1) represent the effect of increasing concentrations of each compound on embryonic mortality, while graphs (2) show the corresponding survival rates. Treatments with molecule 2a and Bz demonstrate dose-dependent variations in embryonic viability.

In the dose-response assay (Fig. 7A-1), the sigmoid curve demonstrated a direct relationship between concentration and mortality, with an LC50 calculated at 14.56 µM. This value indicates the concentration required to induce 50% lethality in the tested population, serving as a toxicological reference parameter. The curve also revealed a behaviour with a steep transition range, suggesting that small variations in dose result in significant changes in mortality.

In the time-related survival assay (Fig. 7A-2), it was evident that the toxicity of molecule 2a is time-dependent. At high concentrations, above 50 µM, mortality occurred acutely, with almost complete disappearance of survival in less than 24 h.

However, at doses of 12.5-25 µM, mortality was progressive, increasing gradually until 96 h, which indicates a cumulative effect and possible involvement of metabolic or bioaccumulation mechanisms in the embryo. On the other hand, at concentrations below 6.25 µM, no significant change in survival rate was observed over 96 h, suggesting a safety range for sublethal assays.

The effect of Bz concentration on embryo mortality was also determined. The LC50 value was 14.7 µM, corresponding to the median lethal concentration, with a 95% confidence interval (CI) between 13.7 µM and 15.8 µM (graph B and A).

Furthermore, the time-related effect on the survival rate of ZebraFish embryos at different concentrations was also evaluated. It was observed that the higher the concentration, the lower the survival rate over time, indicating a dose- and time-dependent effect.

## DISCUSSION

Recently, our research group described the activity of 1,2,4-oxadiazole derivatives against tumour cell lines and their possible immune-mediated mechanisms through a systematic review.^18^ Furthermore, it evaluated their antitumour and immunomodulatory effects in melanoma cells,^19^ and explored their leishmanicidal^20^ and trypanocidal activities *in vitro*.^12^

Oxadiazoles are five-membered heterocyclic compounds that have been widely explored in various scientific areas, such as the pharmaceutical industry, in drug discovery, for example.

Among the known isomers, the one of greatest interest is 1,2,4-oxadiazole due to its diverse biological activities, such as anti-inflammatory,^21^ anticancer,^22^ anticonvulsant^23^ and antiparasitic.^24^

The three oxadiazole derivatives (2a, 2f and 2i) prepared here were evaluated *in vitro* against the epimastigote, trypomastigote and replicative amastigote forms of *T. cruzi* and *in vivo* (2a) to evaluate their cytotoxic effect. First, we used the Y strain of *T. cruzi*, classified for its high *in vitro* infectivity and partial resistance to Bz, in addition to constituting an important research model.^25^ ^,26^

Initially, the cytotoxic effect of the study molecules was evaluated in host cells, which revealed differences in the cellular toxicity profile when compared. The LLC-MK2 cell line (monkey renal epithelial cells) is widely used in *in vitro* studies of *T. cruzi* precisely because of its high susceptibility to infection, viability in culture and ease of laboratory manipulation.^27^

The oxadiazole derivatives caused a statistically significant reduction in cellular function compared to the control groups. Furthermore, host cell toxicity was also measured by estimating the concentration required to reduce host cell viability by 50% (CC50).

According to the results, Bz presented an estimated CC50 of 59.1 µM, at which it is possible to observe moderate toxicity, probably due to its structure, with a nitroimidazole group (-NO2), known in the literature for generating ROS after its bioactivation, affecting host cells.

The compounds 2f (CC50 14 µM) and 2i (CC50 25.7 µM) are lipophilic and electronegative molecules, containing trifluoromethyl (-CF3) and fluorine (-F) groups, respectively.^28^ Both structural behaviours are found in the literature, which reveal that one favours penetration into the cell membrane, but can also affect sensitive organelles and generate cell death (2f) and can affect pharmacokinetic properties and unwanted interactions (2i).^29^

Molecule 2a (CC50 µM) has an unsubstituted phenolic ring linked to a 1,2,4-oxadiazole system. The presence of a cyclohexyl substituent confers a hydrophobic character and increased mass and lipophilicity, characteristics that can reduce cell coverage and the layer by cytotoxic targets, and consequently, contribute to an increase in CC50.^30^

This result confirms the importance of considering the therapeutic window when comparing compounds. Recent data in the literature indicate that a group of pyrazol-imidazoline derivatives also exhibited low toxicity, with CC50 > 100 µM.^31^

Furthermore, there is evidence in the literature that explores the structural effects on the bioactivity and toxicity of the evaluated compounds.

The SI is the ability of a molecule to interact effectively with a desired target compared to an undesired one. SI metrics use the ratio of the IC50s obtained between the targets. The higher the SI, the more selective the molecule of interest.^32^ ^,33^

Our data show that, with the exception of molecule 2f, the other molecules 2a and 2i exhibited high SIs compared to the control group, demonstrating that our molecules have promising potential against *T. cruzi* strains.

Literature data have already demonstrated such activity of molecule 2a in breast cancer and cervical cancer cell lines, which exhibited compound activity at an IC50 of 19.5 μM in MCF-7 cells and 78.7 μM in HeLA cells.^34^ Nevertheless, molecule 2i has also demonstrated activity in tumour lines of lung carcinoma (A549), PC-3 prostate adenocarcinoma, and breast adenocarcinoma (MDA-MB-231), although.^35^

The oxadiazole derivatives presented have as a chemical nucleus a modified hydrazide group (-NH-NH-R) attached to the oxadiazole ring, with structural variations in the substituted phenyl ring.^29^

This structural configuration suggests that these molecules may act as protonophores, that is, weak lipophilic acids capable of transporting protons from the mitochondrial intermembrane space to the matrix, inducing mitochondrial uncoupling.^36^

Furthermore, when analysing the histograms, it is also possible to notice a shift to the right of the fluorescence peaks for both doses, indicating greater DCF intensity. However, no statistically relevant difference was observed between the two concentrations evaluated, suggesting saturation of the pro-oxidant effect.

Performing the same analysis with the 2f molecule, it can be inferred that both concentrations evaluated (50 and 100 μM) promoted a significant increase in cytoplasmic ROS production.

This can be clearly observed in the results obtained by cytometry with depolarisation of the mitochondrial membrane channel. Protonophores utilise the mitochondrial pH gradient to transport protons from the inner membrane space to the mitochondrial matrix.^37^

As a consequence of the electrochemical influx of protons across the inner membrane, there will be a disruption in the supply of the ATP synthase enzyme responsible for ATP synthesis, resulting in a reduction in nutrient metabolism.

The mitochondria will need to increase their metabolic rate to compensate for the gradient leakage, potentially disrupting the parasite's energy-gathering mechanisms and resulting cell death.^38^

In our study, we evaluated the activity of oxadiazole derivatives against the amastigote forms of *T. cruzi*. All molecules demonstrated significant cytotoxic activity, especially when compared to the control treatment with Bz.

One of the main defence mechanisms of amastigote forms is the ability to invade host cells, replicating in the cytoplasm without being recognised, which makes therapies targeting extracellular forms difficult.

Furthermore, transiently dormant and metabolically inactive amastigote forms are resistant to Bz treatment.^39^ As previously mentioned, molecules 2a, 2f, and 2i can undergo changes in their structural conformation, and such changes in their radicals, acting as protonophores, can increase the molecule's lipophilicity.

This property facilitates intracellular penetration and enhances the efficacy of treatment against intracellular parasites.^40^ As already discussed, changes in mitochondrial membrane potential and ROS production were observed in cells treated with molecules 2a, 2f, and 2i.

This effect may be related to the structural nature of the molecules, which can act directly on the mitochondrial membrane; modulation of proton flux may thus influence ROS production.^40^ Excessive ROS production can cause lipid peroxidation of cell membranes, promoting apoptosis and/or late apoptosis, as evidenced in our flow cytometry results.^41^

Simultaneously, in addition to the activities mentioned above, the ability of the molecules to neutralise free radicals in solution was evaluated through the analysis of antioxidant activity using methodologies such as DPPH and ABTS.

Research with new 1,3,4-oxadiazole derivatives also showed antioxidant activity, with an IC50 value of 2.2 µg/mL. From a biological perspective, this observed activity is especially relevant because *T. cruzi* requires a redox system, based on trypanothione reductase and related enzymes, to withstand the oxidative stress imposed by the host.^42^

In this study, molecule 2a showed the highest efficiency, being suggested as a hydrogen donor in the DPPH test, probably due to aromatic conjugation and the presence of NH groups.

However, its activity was limited in the ABTS test, where it showed lower solubility or reduced efficiency in electron donation in aqueous medium.^14^

On the other hand, molecule 2f exhibited a stable antioxidant profile, being more effective in the ABTS test due to the presence of the trifluoromethyl group in its structure, which increases its polarity.

Similarly, molecule 2i showed better performance in the ABTS system, indicating that the structural substituents in these compounds alter their antioxidant capacity according to the type of radical and the physicochemical properties.^43^

These changes are in line with the literature that reports the antioxidant activity of these heterocyclic compounds, which can vary according to the nature of their radical and the physicochemical properties of the molecules. Furthermore, the compounds studied demonstrated a high capacity for scavenging the ABTS radical and reducing the ferric ion.^44^

Furthermore, in the results demonstrated in the evaluation of acetylcholinesterase inhibition, molecule 2a was also greater than the controls, including Bz and the other molecules.

Although *T. cruzi* does not have a functional cholinergic system like mammals, they possess the presence of choline and functional membrane acetylcholine.^45^

Therefore, the inhibition of choline formation could be evaluated using acetylcholinesterase (AChE) enzyme inhibitors. Regarding the ultrastructure of *T. cruzi*, research indicates that the parasite requires specific sterols at all stages of its cell cycle, which ensure cell viability and proliferation.

These sterols, specifically the ergosterol biosynthesis pathway, are essential for the parasite, since they are unable to survive solely on host cholesterol.^46^

Therefore, *T. cruzi* is vulnerable to inhibitors of the 14α-demethylase (CYP51) enzyme, an enzyme that catalyses the conversion of lanosterol to ergosterol, resulting in structural alterations of various organelles and cytotoxic consequences.

Additionally, we employed SEM to evaluate the molecules under study (2a, 2f, and 2i) at concentrations of 25 and 50 µM.

In SEM assay, the molecules (2a, 2f and 2i), at concentrations of 25 and 50 μM, caused different changes in the parasitic forms (epimastigotes), including membrane irregularity, roughness, swelling, cell collapse, and loss of the flagellum. This is comparable to what was observed,^47^ who reported ultrastructural changes in treated trypomastigote forms.

After the *in vitro* assays performed and the results analysed, molecule 2a was an excellent candidate for initiating *in vivo* assays with ZebraFish. The results show that molecule 2a exhibited dose-dependent embryonic toxicity (LC50 14-15 μM).

The use of ZebraFish embryos as an *in vivo* model is validated by their high sensitivity to toxic compounds and their applicability in drug screening. The LC50 obtained (14.56 µM) suggests that molecule 2a exhibits moderate toxicity, with a relatively narrow concentration range between no effect (below 6.25 µM) and significant lethality (above 12.5 µM).

Results in ZebraFish using Bz and molecule 2a were also performed. Molecule 2a demonstrated marked toxicity in *Danio rerio* embryos compared to Bz.

While Bz exhibited the expected dose-dependent profile, with an IC50 of 14.7 µM and mortality at the highest concentrations, molecule 2a caused early mortality at intermediate concentrations, suggesting significant cytotoxic potential.

Even when tested in combination with Bz, the LC50 remained very similar. The chemical nucleus of 2a may contribute to the observed toxicity, while Bz, in this same arrangement, appears to have only a secondary role in acute lethality.

This is consistent with findings in the literature, which report that 1,2,4-oxadiazole derivatives almost frequently exhibit embryonic toxicity in ZebraFish.^30^

Many drug interaction effects only emerge when metabolism is active. However, the embryo has limited metabolic capacity and differences in biotransformation pathways, which may explain the absence of observed interactions.^48^

Further studies are needed to assess how the covariance of toxicity in ZebraFish is influenced by factors such as absorption, metabolism, and toxicity mechanisms. The next step may be to reduce exposure in the organism and increase delivery to the site of action through controlled release, such as loaded nanoparticles, to limit the free availability of the compound.^49^

In conclusion, the molecules studied showed different activities against *T. cruzi*. The mechanism of action was largely related to mitochondrial dysfunction, redox imbalance, and apoptotic processes. Molecule 2a stood out for its great antiparasitic activity *in vitro*, justifying its evaluation in an *in vivo* model, which showed dose-dependent embryonic toxicity. These results provide important information on the therapeutic window and the need for optimisation strategies such as controlled release systems. Further *in vivo* assays in murine infection models are needed, as well as the need for complementary molecular targets.

## Data Availability

The datasets generated and/or analysed during the current study are not publicly available but are available from the corresponding author upon reasonable request.
