# Supplementary material for: Evaluation of the in vitro activity of synthetic derivatives of N-cyclohexyl-3-(3-methylphenyl)-1,2,4-oxadiazole-5-amine on the strain y of Trypanosoma cruzi and an in vivo toxicity study
Source: Mem Inst Oswaldo Cruz. 2026 Jul 20;121(Suppl 2):e250289. doi: 10.1590/0074-02760250289 (PMC13387807; doi:10.1590/0074-02760250289)
Supplement: Peer review history

# Reviewer report: FIRST REVIEW ROUND - REVIEWERS COMMENTS

## REVIEWER #1

Reviewing this manuscript was a pleasure. The work is well-structured and presents interesting findings that may contribute to future alternative therapies for Chagas disease. I have a few minor suggestions for the authors, primarily concerning clarification of terms and improvements to the clarity and flow of the manuscript:

1. The stock concentration values are not described in the Methods section.

2. The acronym MTT should be defined at its first occurrence in the text.

3. The acronym DMEM should be defined at its first occurrence in the text.

4. The origin of the LLC-MK2 cell line is mentioned only in the Discussion; this information should already be provided in the Methods section.

5. Regarding the figure titles, I assumed they followed the order in which they appear. However, in the text you refer to Figures 1, 2, and 3, but the figures themselves are not labeled as such.

6. Please improve the quality of the figures and increase the font size, as even with zooming it was difficult to visualize the data and compare it with what is described in the text.

## REVIEWER #2

This paper investigates the in vitro anti-*Trypanosoma cruzi* activity of three N-cyclohexyl-3-(3-methylphenyl)-1,2,4-oxadiazole-5-amine derivatives (2a, 2f, 2i) across epimastigote, trypomastigote, and intracellular amastigote stages of the Y strain, and explores putative death mechanisms via flow cytometry (Annexin V/7-AAD, DCFH-DA, Rh123) and SEM. It also assesses in vivo acute embryotoxicity of the most selective compound (2a) in zebrafish embryos under OECD FET236. The work addresses an important unmet need in Chagas disease drug discovery, where current therapies are limited, particularly regarding chronic infection. It provides starting points for structure–activity–toxicity (SAT) considerations within the oxadiazole scaffold class and flags embryotoxicity risks early in the process.

Please replace the "Conclusion" section with "Results" in the abstract.

Introduction: When mentioning the biological activities of the 1,2,4-oxadiazole class in the final paragraph of the introduction, the authors should include its antiparasitic activity (as stated in the abstract). The specific objective of the study must also be clearly stated in this final paragraph.

Results and Discussion: The study identifies compound 2a as the most selective and 2i as the most potent. Consider to perform functional assays (ATP quantification/mitochondrial respiration) and include positive controls to calibrate the flow cytometry readings and gating to validate the proposed mechanism of action.

Efficacy against amastigotes is critical for translational relevance. Provide a complete dose-response curve (at least 6 concentrations), IC50 values and percentage of infected cells.

Consider to expand host cell cytotoxicity assays to include human-relevant cells (derived cardiomyocytes or human fibroblasts) to better estimate the therapeutic index in disease-relevant tissues.

The Conclusion section must be integrated as part of the main text.

The manuscript needs revision for English.

Verify and ensure correct units, labels, and legends for all tables and graphs.

Avoid using abbreviations when referring to Figures and Tables within the text. Standardize the format using title case (e.g., Figure 1, Table 2).

Titles and Resolution: All figures are missing titles. Figures 2, 3, 4, and 5 are in low resolution, making it impossible to read the graphs and legends.

Table 1: Change the numbering from "Table I" to "Table 1".

Units: Include the concentration unit for the CC50 (LLC-MK2) values.

## AUTHORS' RESPONSE TO THE REVIEWERS

February, 14th, 2026.

Dear Editor,

I am sending the revised form of the manuscript entitled "*EVALUATION OF THE IN VITRO ACTIVITY OF SYNTHETIC DERIVATIVES OF N-CYCLOHEXYL-3(3-METHYLPHENYL)-1,2,4-OXADIAZOLE-5AMINE ON THE STRAIN Y OF Trypanosoma cruzi AND AN IN VIVO TOXICITY STUDY*" to be considered for publication in the "MIOC".

The suggestions made for this revision have been incorporated in the text. We trust that corrections and additions made to the manuscript have improved it considerably and we hope that you will now find it suitable for publication.

We would like to thank you for the interest by our study.

The specific criticisms and suggestions made to this study will be pointed below and changes in the manuscript were also highlighted, as indicated:

In response to the reviewers' comments:

## Reviewer #1

Reviewing this manuscript was a pleasure. The work is well-structured and presents interesting findings that may contribute to future alternative therapies for Chagas disease. I have a few minor suggestions for the authors, primarily concerning clarification of terms and improvements to the clarity and flow of the manuscript:

R: Thanks for your interest and suggestions made for improving the manuscript!

**1. The stock concentration values are not described in the Methods section.**

It was inserted, as required (page 2, section Chemicals).

**2. The acronym MTT should be defined at its first occurrence in the text.**

It was inserted, as required (page 2, section Cytotoxicity Assay).

**3. The acronym DMEM should be defined at its first occurrence in the text.**

It was inserted, as required (page 2, section Cytotoxicity Assay).

**4. The origin of the LLC-MK2 cell line is mentioned only in the Discussion; this information should already be provided in the Methods section.**

It was inserted, as required (page 2, section Cytotoxicity Assay).

**5. Regarding the figure titles, I assumed they followed the order in which they appear. However, in the text you refer to Figures 1, 2, and 3, but the figures themselves are not labeled as such.**

It was corrected, as required for the entire text.

**6. Please improve the quality of the figures and increase the font size, as even with zooming it was difficult to visualize the data and compare it with what is described in the text.**

Thanks! All figures were improved, as required.

## Reviewer #2

This paper investigates the in vitro anti-*Trypanosoma cruzi* activity of three N-cyclohexyl-3-(3-methylphenyl)-1,2,4-oxadiazole-5-amine derivatives (2a, 2f, 2i) across epimastigote, trypomastigote, and intracellular amastigote stages of the Y strain, and explores putative death mechanisms via flow cytometry (Annexin V/7-AAD, DCFH-DA, Rh123) and SEM. It also assesses in vivo acute embryotoxicity of the most selective compound (2a) in zebrafish embryos under OECD FET236. The work addresses an important unmet need in Chagas disease drug discovery, where current therapies are limited, particularly regarding chronic infection. It provides starting points for structure–activity–toxicity (SAT) considerations within the oxadiazole scaffold class and flags embryotoxicity risks early in the process.

**Please replace the "Conclusion" section with "Results" in the abstract.**

Thanks! It was corrected, as required (page 1, highlighted in the text).

**Introduction: When mentioning the biological activities of the 1,2,4-oxadiazole class in the final paragraph of the introduction, the authors should include its antiparasitic activity (as stated in the abstract). The specific objective of the study must also be clearly stated in this final paragraph.**

It was added in the text, as required (page 2, highlighted in the Introduction section).

**Results and Discussion: The study identifies compound 2a as the most selective and 2i as the most potent. Consider to perform functional assays (ATP quantification/mitochondrial respiration) and include positive controls to calibrate the flow cytometry readings and gating to validate the proposed mechanism of action.**

Thanks for the suggestion to expand our assays. In this study was conducted an antioxidant assay and also was included now the acetylcholinesterase inhibition assay to reinforce the *in vitro* effects of the compounds tested and better speculate the differences between each one derivative regarding distinct targets on the parasite. We added the methodology and also better discussed information regarding these mechanisms, especially for 2a molecule, in the revised text highlighted in the pages 4, 6 and 8 and also in the results from Table 2. Moreover, a discussed text correlating ROS inhibition and mitochondrial activity was already pointed in the Discussion section (pages 7 and 8): "Performing the same analysis with the 2f molecule, it can be inferred that both concentrations evaluated (50 and 100 μM) promoted a significant increase in cytoplasmic ROS production.

This can be clearly observed in the results obtained by cytometry with depolarization of the mitochondrial membrane channel. Protonophores utilize the mitochondrial pH gradient to transport protons from the inner membrane space to the mitochondrial matrix (37).

As a consequence of the electrochemical influx of protons across the inner membrane, there will be a disruption in the supply of the ATP synthase enzyme responsible for ATP synthesis, resulting in a reduction in nutrient metabolism.

The mitochondria will need to increase their metabolic rate to compensate for the gradient leakage, potentially disrupting the parasite's energy-gathering mechanisms and resulting cell death (38)"

Regarding the flow cytometry assay, the positive control of the gating strategy in the 7aad and annexin v experiment is justified by the robustness of the marker, as you observe in the graph. Some articles also do not use this positive control. Although they do not use a known agent to induce apoptosis as a positive control, they compare biological states (apoptotic vs. non-apoptotic) in real populations. For Ros production and **ΔΨ**m determination the positive controls were described in the text (highlighted in pages 3 and 4). We have also performed our assay in line with this similar approach, e.g by the manuscripts listed below and highlighted in the References section:

-ARAÚJO, Héverton Mendes et al. In vitro antitumor and immunomodulatory activities of 1, 2, 4-oxadiazole derivatives. Biochemistry and Biophysics Reports, v. 41, p. 101950, 2025.

-DE, Pradip et al. Triple fluorescence staining to evaluate mechanism-based apoptosis following chemotherapeutic and targeted anti-cancer drugs in live tumor cells. Scientific reports, v. 8, n. 1, p. 13192, 2018.

-ANTHONY, R. S. et al. Flow cytometry using annexin V can detect early apoptosis in peripheral blood stem cell harvests from patients with leukaemia and lymphoma. Bone marrow transplantation, v. 21, n. 5, p. 441-446, 1998.

**Efficacy against amastigotes is critical for translational relevance. Provide a complete dose-response curve (at least 6 concentrations), IC50 values and percentage of infected cells.**

Thank you for your suggestion! We agree that an IC50 determination requires a full dose-response curve with multiple concentrations spanning the dynamic range. In the intracellular amastigote assay conducted here, only two concentrations (30 and 60 µM) were tested due to mainly previous standardization of these concentrations employed in previous assays from our research group and also to experimental limitations at the time of the study, which prevents a robust logistic fit of 4 more parameters. This is in line with published guidelines indicating that a true IC50 prediction requires ≥5 spanning the most response range and multiple concentrations above and below 50% effect.

To meet your request properly, we report (i) the percentage of infected cells at each concentration and (ii) the normalized parasite load (amastigotes per 100 cells) expressed as % survival relative to the untreated control (Figure 2). In addition, we provide an IC50 estimated by logarithmic interpolation between 30 and 60 µM (IC50 ≈ 72 µM), clearly labeled. This is an approximation due to the limited number of concentrations. The complete dose-response curve (≥6 concentrations) will be developed for further studies.

**Consider to expand host cell cytotoxicity assays to include human-relevant cells (derived cardiomyocytes or human fibroblasts) to better estimate the therapeutic index in disease-relevant tissues.**

We appreciate the reviewer's suggestion regarding the inclusion of human-relevant host cells (e.g., human fibroblasts or cardiomyocytes) to better estimate the tissue-related therapeutic index. In this study, cytotoxicity was evaluated in LLC-MK2 cells because (i) LLC-MK2 is a highly permissive, non-phagocytic epithelial host cell widely used for *T. cruzi* propagation and intracellular amastigote assays, allowing for robust and reproducible infection readings, and (ii) using the same host cell line as the intracellular assay allows for a consistent *in vitro* selectivity index (CC50/IC50), minimizing interference from host cell death. It is important to emphasize that the choice of host cell influences *T. cruzi* susceptibility profiles and, therefore, any *in vitro* model represents a balance between productivity/reproducibility and tissue specificity. We would like to clarify that in future work we will expand the toxicity analysis to relevant human cells in order to refine the estimates of the translational therapeutic index. Please, see some articles employing the LLC-MK2 cells, as in our study (highlighted in the References section):

-BETTIOL, Esther et al. Identification of three classes of heteroaromatic compounds with activity against intracellular *Trypanosoma cruzi* by chemical library screening. PLoS neglected tropical diseases, v. 3, n. 2, p. e384, 2009.

-DE MENEZES, Ramon RPPB et al. Antiparasitic effect of (−)-α-bisabolol against *Trypanosoma cruzi* Y strain forms. Diagnostic microbiology and infectious disease, v. 95, n. 3, p. 114860, 2019.

-PITASSE-SANTOS, Paulo et al. A novel protocol for the synthesis of 1, 2, 4-oxadiazoles active against trypanosomatids and drug-resistant leukemia cell lines. Tropical Medicine and Infectious Disease, v. 7, n. 12, p. 403, 2022.

**The Conclusion section must be integrated as part of the main text.**

It was adjusted, as pointed it (highlighted in page 9). Thanks!

**The manuscript needs revision for English.**

It was checked and improved it.

**Verify and ensure correct units, labels, and legends for all tables and graphs.**

It was checked for the entire manuscript.

**Avoid using abbreviations when referring to Figures and Tables within the text. Standardize the format using title case (e.g., Figure 1, Table 2).**

It was corrected in the entire manuscript (highlighted).

**Titles and Resolution: All figures are missing titles. Figures 2, 3, 4, and 5 are in low resolution, making it impossible to read the graphs and legends.**

It was corrected for all figures. Thanks!

**Table 1: Change the numbering from "Table I" to "Table 1".**

It was corrected (highlighted in page 5).

**Units: Include the concentration unit for the CC50 (LLC-MK2) values.**

It was corrected (highlighted in page 2, section Cytotoxicity Assay).

Thank you for your attention and valuable contributions!

Roberto Nicolete, PhD

# Reviewer report: REVIEWERS COMMENTS

## REVIEWER #1

No comments

## REVIEWER #2

No comments
